# An Unusual Presentation of Primary Hepatic Diffuse Large B-cell Lymphoma of the Liver

**DOI:** 10.7759/cureus.2242

**Published:** 2018-02-27

**Authors:** Rohit Dhingra, Michael W Winter, Osman H Yilmaz, Sunny Jaiswal, Mark Sterling

**Affiliations:** 1 Department of Medicine, Tufts Medical Center; 2 Division of Gastroenterology, Tufts MedicalCenter; 3 Department of Pathology, Tufts Medical Center; 4 Department of Radiology, Tufts Medical Center; 5 Division of Gastroenterology, Tufts Medical Center

**Keywords:** lymphoma, liver tumors, diffuse large b cell lymphoma, hepatology

## Abstract

This report describes a case of primary hepatic diffuse large B-cell lymphoma (DLBCL) in a 64-year-old male who presented with constitutional symptoms, jaundice, abdominal swelling, and right upper quadrant pain. The diagnosis was confirmed on percutaneous liver biopsy. Notably, there was no evidence of extra-hepatic involvement. The patient received methylprednisolone and cyclophosphamide with good response but was lost to follow-up upon being transferred. This case highlights the importance of considering primary hepatic DLBCL in patients with unexplained abnormal liver tests and atypical imaging without solitary or discrete lesions, as this rare malignancy can present furtively.

## Introduction

Primary hepatic diffuse large B-cell lymphoma (DLBCL) is an uncommon type of non-Hodgkin’s lymphoma, accounting for ~0.4% of extra-nodal non-Hodgkin’s lymphoma, and it rarely presents without spread to the lymph nodes, bone marrow, or other organs [1]. The etiology of primary hepatic DLBCL is unknown. Immune suppressed patients are more susceptible, yielding hypotheses that certain viral (Epstein-Barr virus, hepatitis B, hepatitis C, human immunodeficiency virus) or autoimmune conditions may promote development of this rare cancer. Since it presents with non-specific symptoms and inconclusive radiographic findings, a high index of clinical suspicion and low threshold to obtain a targeted biopsy is critical to confirming the diagnosis of primary hepatic DLBCL.

## Case presentation

**Figure 1:**
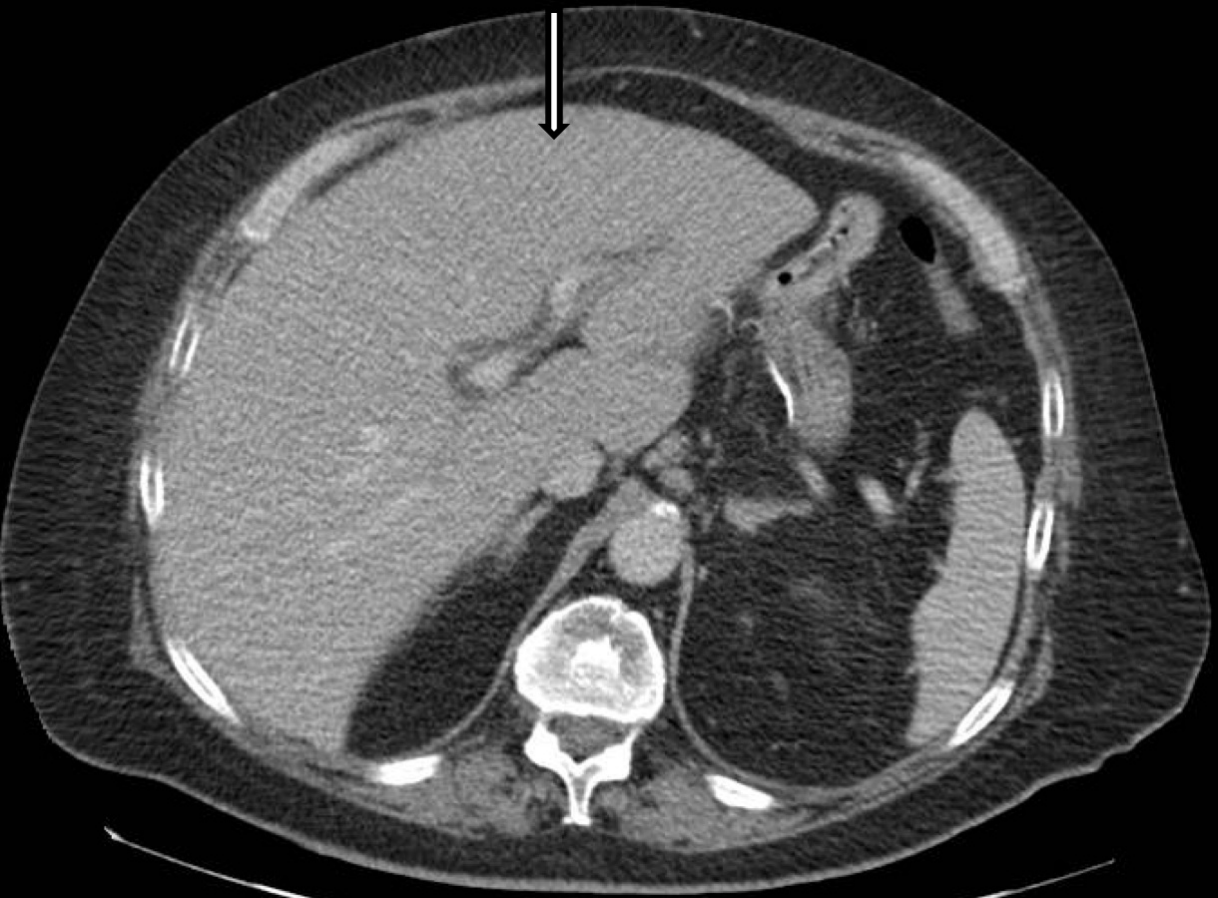
Computed Tomography (CT) of the Abdomen with Intravenous contrast Axial image at the level of the liver demonstrates a mildly enlarged liver without a discrete focal mass (arrow)

A 64-year-old male with a past medical history of morbid obesity, Roux-en-Y gastric bypass, cholecystectomy, type II diabetes mellitus, and hypertension presented with lower extremity and abdominal swelling, right upper quadrant pain, jaundice, myalgia, and fatigue. He presented to his primary care physician three weeks prior with myalgia, lower extremity swelling, and fatigue. His lab notable test results were elevated aspartate aminotransferase (AST; 99 IU/L), alanine aminotransferase (ALT; 106 IU/L), and alkaline phosphatase (AP; 135 IU/L). Computed tomography (CT) of the abdomen / pelvis with intravenous contrast demonstrated circumferential thickening of the distal esophagus with small adjacent peri-esophageal lymph nodes that are nonspecific with a mildly enlarged liver without a discrete focal mass (Figure 1).

**Figure 2:**
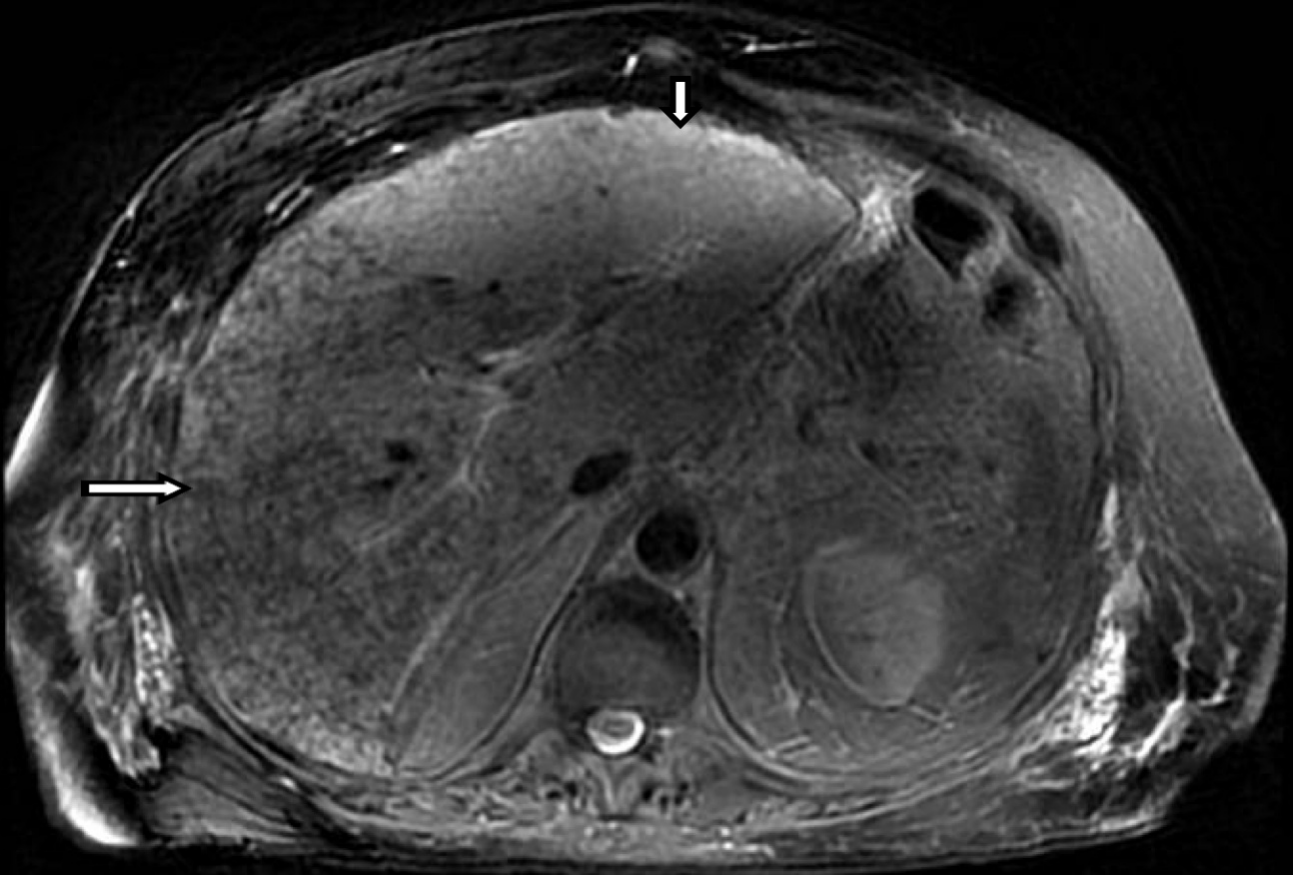
Magnetic Resonance Imaging (MRI) of the Abdomen without contrast T2 weighted axial image at the same level of the liver demonstrates abnormal heterogeneous increased T2 signal predominately involving the left hepatic lobe (small arrow). There are small areas of normal liver seen (long arrow), suggesting an infiltrative process.

He presented to our institution with right upper quadrant pain and abdominal swelling with constitutional symptoms and lower extremity swelling. His physical examination was notable for scleral icterus, hepatosplenomegaly, and right upper quadrant tenderness. Laboratory findings showed a leukocytosis, worsened liver function (AST: 234 IU/L, ALT: 118 IU/L, ALP: 358 IU/L), total bilirubin elevation (TB; 1.6 mg/dL), lactate 4.3 MEQ/L, albumin 2.4 g/dL. Magnetic resonance cholangiopancreatography (MRCP) demonstrated abnormal heterogeneous increased T2 signal predominantly involving the left hepatic lobe with small areas of normal liver seen, suggesting an infiltrative process (Figure 2).

**Figure 3:**
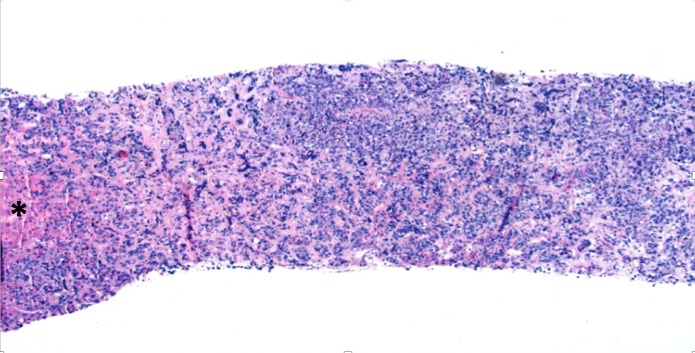
Liver Core Biopsy Liver core biopsy extensively involved by lymphoid cells with sparse hepatic parenchyma (*)

**Figure 4:**
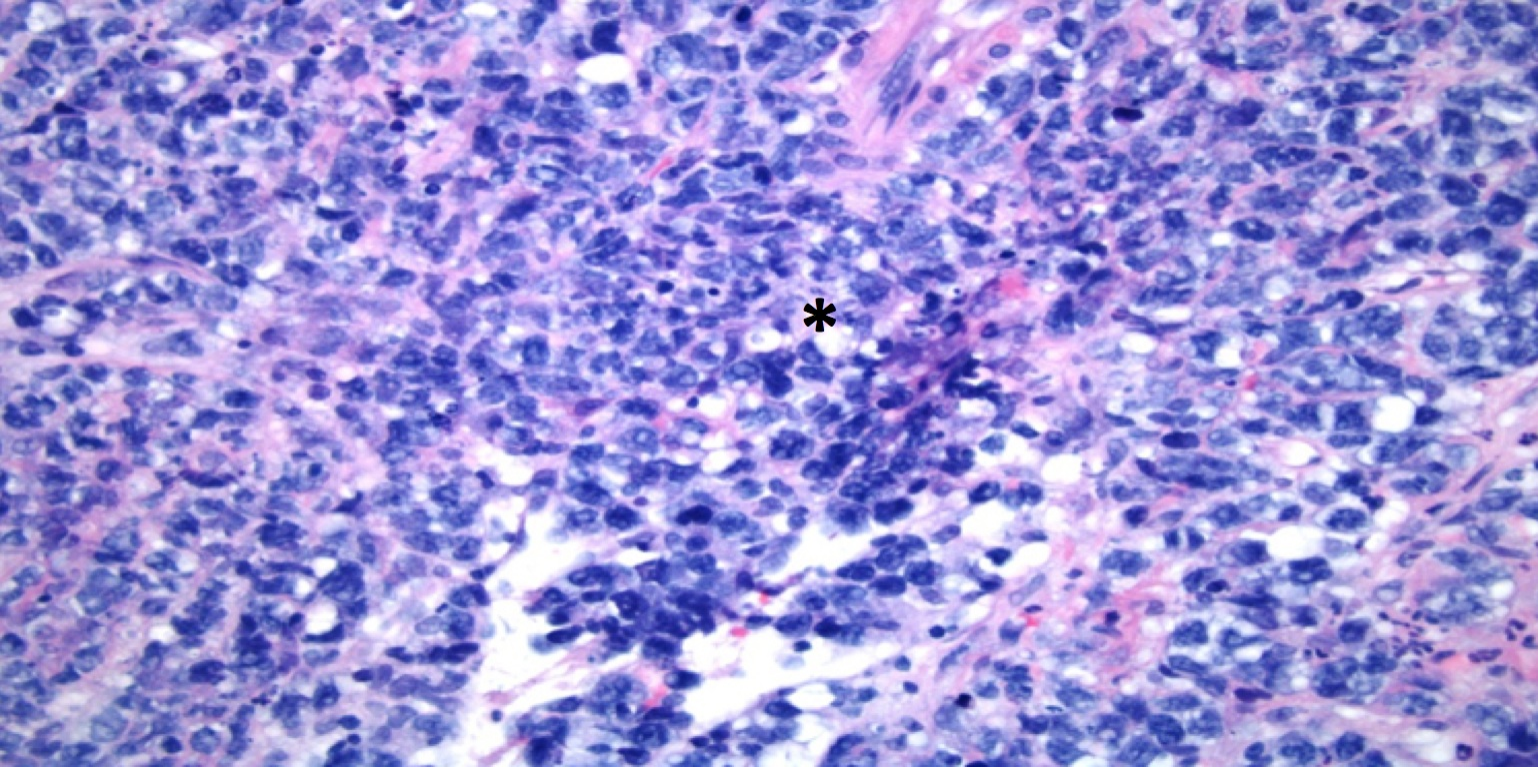
Higher Magnification of Liver Biopsy Higher magnification of this diffuse infiltrate (*) reveals frequent morphological characteristics of DLBCL including large sized lymphoid cells with irregular nuclei, vesicular chromatin, small nucleoli, and moderate amount of cytoplasm.

**Figure 5:**
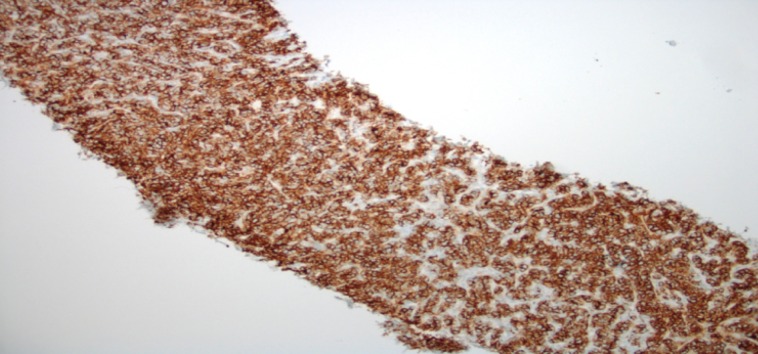
Liver Core Biopsy with CD-20 Immunohistochemical Staining Immunohistochemical staining reveals the atypical lymphoid cells to be diffusely positive for CD20.

**Figure 6:**
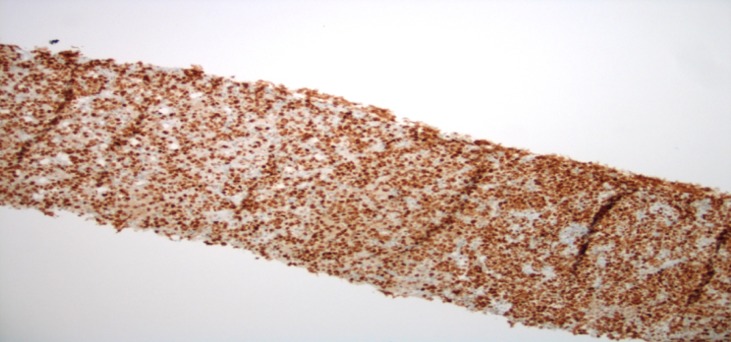
Liver Core Biopsy with bcl-6 Immunohistochemical Staining Liver Core Biopsy with bcl-6 Immunohistochemical Staining

There was no evidence of cholestasis on hepatobiliary iminodiacetic acid (HIDA) scan or biliary obstruction on endoscopic retrograde cholangio-pancreatography (ERCP). There were no malignant cells identified on peri-esophageal lymph node biopsy. Hepatitis virus A/B/C, Epstein-Barr virus, cytomegalovirus, rickettsial, autoimmune, iron, carcinoembryonic antigen serum, and calcium serologies were normal with the absence of any other signs of paraneoplastic phenomena. Our patient had worsening elevation in his liver function tests (total bilirubin: 4.1 mg/dL, direct bilirubin: 3.1 mg/dL). A non-targeted percutaneous liver biopsy showed to be extensively involved by lymphoid cells with sparse hepatic parenchyma (Figure 3). Higher magnification revealed morphological characteristics of DLBCL (Figure 4) and immunohistochemical staining revealed atypical lymphoid cells to be diffusely positive for CD-20 (Figure 5) and bcl-6 (Figure 6). Flow cytometry showed that, of the total cells, differentiating myeloid cells represented the majority composing of 87%, T cells 3% (CD4:CD8: ratio was 2:1), and B-cells <1% (polytypic).

Collectively, this immunomorphologic phenotype supports a diagnosis of DLBCL. Bone marrow aspirate did not reveal any malignant cells. He was evaluated by an oncologist at our institution and was initially only treated with intravenous (IV) cyclophosphamide and methylprednisone given that rituximab and doxorubicin (ie R-CHOP) was contraindicated with his current worsening liver function and elevated bilirubin. The treatment plan was to treat him with rituximab, cyclophosphamide, doxorubicin, vincristine, and prednisone (R-CHOP) pending improvement in his liver function. However, the patient chose to seek a second opinion and was lost to follow-up.

## Discussion

Primary hepatic DLBCL without extrahepatic spread is a rare malignancy, particularly in patients without underlying immunosuppression or chronic liver disease [2]. This case highlights the importance of considering this diagnosis in patients presenting with nonspecific symptoms and abnormal liver function tests even in the absence of a discrete liver lesion. Imaging findings for primary hepatic DLBCL are variable, demonstrating either a solitary (42%) or multiple (50%) lesions in the majority of known cases [3]. Our patient’s presentation underscores the importance of considering DLBCL in the absence of solitary or several hepatic lesions, as neither CT nor MRCP revealed discrete lesions in this instance. The differential diagnosis must remain broad and should always include, in addition to lymphoma, other infiltrative processes of the liver including metastatic malignancy, sarcoidosis, tuberculosis, and hemochromatosis [4]. Klatskin tumor or hilar cholangiocarcinoma should also be considered in a patient with this presentation as an obstruction at the junction of the left and right hepatic ducts can cause jaundice and acute liver failure [5]. Liver biopsy is necessary to confirm the diagnosis of DLBCL, in addition to the absence of lymphoma in the bone marrow and lymph nodes. Treatment options for DLBCL include surgical resection, chemotherapy, and in some instances, radiation therapy. The overall prognosis is poor, and the median overall survival for these patients has been estimated to be approximately 33 months [6]. Given that affected patients may present with nonspecific symptoms with non-revealing findings on imaging, DLBCL can often be a diagnostic challenge. Unfortunately, DLBCL is often diagnosed in advanced disease where therapeutic interventions are of minimal benefit. This case highlights the importance of pursuing a liver biopsy early in the workup for abnormal liver tests of an unclear etiology, even in the absence of a liver lesion on imaging, as in some instances a more expedited diagnosis can improve prognosis.

## Conclusions

Our case describes a rare type of DLBCL without extrahepatic involvement. It is important to consider this diagnosis along with other types of infiltrative disease processes and pursue targeted liver biopsy in patients presenting with nonspecific symptoms, abnormal liver function tests, and absence of either solitary or multiple liver lesions without an alternative explanation.
